# 

**Published:** 1907-08

**Authors:** 


					﻿Vol. XXIIL_
AUGUST, 1907.
No. 2?
TEXAS
MEDICAL
JOURNAL
“RED BACK”
PUBLISHED AT AUSTIN, TEXAS, BY
F. E. DANIEL, M. D.,	-	-
PRINTED BY THE VON BOECKMANN-JONES (Xi, *
Subscription, $1.00 a Year, in Advance. ... Single Copies, 10 Cents
Entered at the Postoffice at Austin, Texas, as Second-class Mail Matter
				

